# Association between C-reactive protein-albumin-lymphocyte (CALLY) index and atrial fibrillation recurrence: A retrospective cohort study

**DOI:** 10.1097/MD.0000000000049012

**Published:** 2026-05-22

**Authors:** Xiaojian Zhang, Yanping Yin, Qingru Zhu, Sixiang Jia, Weili Ge, Shudong Xia

**Affiliations:** a Department of Cardiology, the Fourth Affiliated Hospital of School of Medicine, and International School of Medicine, International Institutes of Medicine, Zhejiang University, Yiwu, China; b Department of Cardiology, Taizhou Hospital of Zhejiang Province affiliated to Wenzhou Medical University, Linhai, Zhejiang Province, China; c Laboratory of Cardiovascular Disease, Taizhou Hospital of Zhejiang Province affiliated to Wenzhou Medical University, Linhai, Zhejiang Province, China.

**Keywords:** atrial fibrillation recurrence, CALLY Index, radiofrequency catheter ablation

## Abstract

The C-reactive protein-albumin-lymphocyte (CALLY) index reflects the body’s inflammatory, nutritional, and immune status, and has been shown to correlate with cancer prognosis. However, its impact on atrial fibrillation (AF) ablation outcomes remains unclear. We retrospectively analyzed 1042 AF patients who underwent catheter-based radiofrequency catheter ablation (RFCA) at 2 tertiary hospitals in China: The Fourth Affiliated Hospital of Zhejiang University School of Medicine and Taizhou Hospital of Zhejiang Province. Each patient was followed for at least 3 months. AF recurrence was defined as the occurrence of atrial arrhythmias (atrial tachycardia, atrial flutter, or atrial fibrillation) lasting longer than 30 seconds after RFCA. Cox regression was used to explore the association between the CALLY index and AF recurrence, while restricted cubic spline (RCS) plots were employed to elucidate the relationship. Receiver operating characteristic curves were generated to compare the predictive value of the CALLY index with traditional immune-inflammatory markers: the Systemic Immune-Inflammation Index, platelet/high-density lipoprotein ratio, and neutrophil/high-density lipoprotein ratio. Among the 1042 patients who underwent AF RFCA, the median age was 57 years (interquartile range [IQR], 57–70 years). A total of 211 patients (20.25%) experienced AF recurrence, with a median follow-up duration of 27.00 months (IQR, 17.00–42.00 months). The median CALLY index was 5.22 (IQR, 2.11–10.72). After adjusting for confounding factors, the CALLY index was found to be a protective factor for AF recurrence. For each 1-unit increase in the CALLY index, the hazard of AF recurrence decreased by 10% (adjusted hazard ratio = 0.8974, 95% confidence interval [CI]: 0.8674–0.9284, *P* < .001). RCS plots revealed a nonlinear association between the CALLY index and AF recurrence (*P* overall < .001). The CALLY index demonstrated superior predictive value compared to traditional immune-inflammatory markers (AUC = 0.667, 95% CI: 0.628–0.706). The CALLY index before AF ablation is associated with AF recurrence outcomes. It provides a comprehensive reflection of the pre-ablation inflammatory, nutritional, and immune status in AF patients and demonstrates better predictive value compared to traditional immune-inflammatory markers.

## 1. Introduction

Atrial fibrillation (AF) is a common arrhythmia in clinical practice. Radiofrequency catheter ablation (RFCA) serves as a first-line treatment for AF; however, post-ablation recurrence rates remain high.^[1,2]^

Multiple factors contribute to recurrence after AF ablation, including inflammation, nutritional status, and immune function.^[3]^ In AF patients, the inflammatory response promotes atrial structural remodeling, leading to alterations in the atrial matrix and mediating electrical remodeling, both of which can diminish ablation efficacy.^[4]^ Researchers such as Zhu S and Badheka AO have identified nutritional status as a risk factor for AF recurrence.^[5,6]^ Nutritional imbalance can compromise protein and caloric intake, activating pro-inflammatory pathways and thereby reducing the success rate of AF ablation. Growing evidence also points to a connection between immune responses and AF, suggesting that short-term immune modulation early in AF management may interrupt the inflammatory cycle and mitigate adverse outcomes in patients undergoing ablation therapy.^[7]^ Hence, it is essential to identify a comprehensive indicator that reflects inflammation, nutrition, and immune function, enabling better assessment of AF patients’ overall condition and facilitating early intervention to lower recurrence rates.

The C-reactive-protein-albumin-lymphocyte (CALLY) index, introduced by Hiroya Iida et al., is an emerging parameter that integrates information on inflammation, nutrition, and immune status.^[8]^ While it has been associated with survival outcomes in cancer patients, its predictive value for AF ablation prognosis remains unclear.^[9]^ This study aims to investigate the prognostic significance of the CALLY index in patients undergoing AF RFCA and to compare it with established immune-inflammation indices widely used in clinical practice, such as the Systemic Immune-Inflammation Index (SII), platelet/high-density lipoprotein ratio (PHR), and neutrophil/high-density lipoprotein ratio (NHR).^[10,11]^

## 2. Methods

### 2.1. Study population

This retrospective cohort study enrolled 1372 patients scheduled for AF RFCA from 2 top-tier hospitals in China: the Fourth Affiliated Hospital of Zhejiang University School of Medicine, Taizhou Hospital of Zhejiang Province. AF Patients’ information was exported through the Hospital Information System (HIS).

Exclusion criteria were as follows:

1. Loss to follow-up post-ablation.
2. Multiple repeated ablations.
3. Patients with missing pre-ablation data on CRP, albumin, and lymphocyte count.
4. Patients with rheumatic heart valve disease, thyroid dysfunction, severe heart failure, or malignancies.

**Figure 1.**
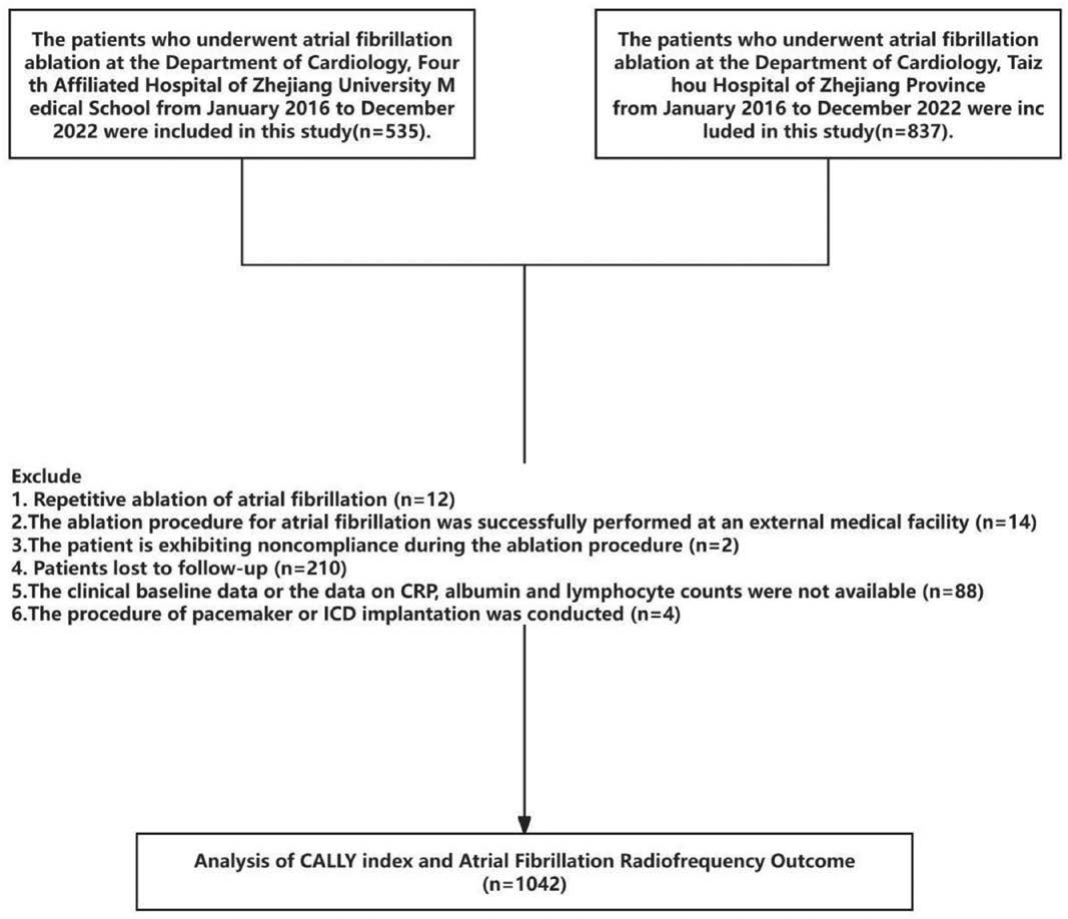
Flow chart of this study.

The ablation procedure and postoperative follow-up details are provided in the Supplementary A1 and A2. Based on these criteria, 1042 patients were ultimately included in the study. The follow-up period for this study extends until December 2023. This study was approved by the Ethics Committee of the Fourth Affiliated Hospital of Zhejiang University School of Medicine (NO.K2025027) as conducted in accordance with the guidelines of the Declaration of Helsinki. As this is a retrospective study, informed consent from patients was waived. Figure 1 shows the flow chart of this study.

### 2.2. Definition of atrial fibrillation recurrence

According to the latest ACC/AHA guidelines, AF recurrence is defined as the presence of atrial arrhythmias (atrial tachycardia, atrial flutter, or atrial fibrillation) lasting longer than 30 seconds after the RFCA. Recurrences occurring within 3 months postoperatively are classified as early recurrences, while those occurring after 3 months are considered late recurrences.^[1]^

### 2.3. Patient characteristics

Data on the following demographic and clinical characteristics were collected from patients undergoing atrial fibrillation ablation prior to the procedure: gender, age, height, weight, type of AF, left atrial diameter, left ventricular ejection fraction (LVEF), hypertension, diabetes, coronary artery disease, use of antiarrhythmic medications, neutrophil count, lymphocyte count, platelet count, serum albumin level, C-reactive-protein (CRP) level, serum creatinine (Scr) level, estimated glomerular filtration rate (eGFR), total cholesterol (TC) level, triglyceride level, high-density lipoprotein cholesterol (HDL-C) level, low-density lipoprotein cholesterol (LDL-C) level, glucose level, glycated hemoglobin (HbA1c) level. Additionally, the triglyceride-glucose (TyG) index, CHA2DS2-VASc score, and HAS-BLED score were calculated, as detailed in the Supplementary A3.

### 2.4. Calculating the CALLY Index, SII, NHR, PHR, and LHR

CALLY index = 0.1 × Albumin (g/L) × Lymphocyte Counts (10^9^/L) ÷ CRP (mg/L)

SII = Platelet counts (10^9^/L) × neutrophil counts (10^9^/L) ÷ Lymphocyte counts (10^9^/L)

NHR = Neutrophil counts (10^9^/L) ÷ HDL-C (mmol/L)

PHR = Platelet counts (10^9^/L) ÷ HDL-C (mmol/L)

Lymphocyte/high-density lipoprotein ratio (LHR) = Lymphocyte counts (10^9^/L) ÷ HDL-C(mmol/L)

### 2.5. Statistical analysis

Data are presented as simple percentages or medians within quartile ranges. Fisher exact test or chi-square test was used to evaluate baseline characteristics. The Student *t* test was applied for continuous variables with a normal distribution, while the Mann–Whitney U test was used for continuous variables with a non-normal distribution. Missing values (<30%) were imputed using the “mice” package in R.

We first examined the correlation between the CALLY index and traditional immune-inflammatory markers, including SII, NHR, PHR, and LHR, and visualized the results using a heatmap. Univariate Cox regression was employed to identify risk factors associated with AF recurrence, followed by multivariate Cox regression models to compare the associations between CALLY and traditional systemic immune-inflammatory markers (SII, NHR, PHR, LHR) with AF recurrence. Model 1 did not adjust for confounders, Model 2 adjusted for age, gender, and BMI, and Model 3 further adjusted for AF type, AF duration, NT-proBNP, triglycerides, left atrial diameter, LVEF, HbA1c, TyG index, glucose, and the use of class II antiarrhythmic drugs.

To further clarify the relationship between CALLY and AF recurrence, we constructed restricted cubic spline (RCS) curves to explore their association, adjusting for confounders. Subgroup analyses were conducted to investigate potential heterogeneity of CALLY in relation to AF recurrence across various subgroups, including age (cutoff: 65 years), sex, left atrial diameter (cutoff: 38 mm), and the presence of hypertension, diabetes, coronary artery disease, and heart failure (LVEF cutoff: 50%). Forest plots were generated to illustrate these subgroup differences. To assess the predictive value of CALLY for AF recurrence, we generated receiver operating characteristic (ROC) curves. A *P* value <0.05 was considered statistically significant, all statistical analyses were performed using R version 4.3.1.

## 3. Results

### 3.1. Patient characteristics

Among the 1042 patients who underwent AF RFCA, the median age was 57 years (interquartile range [IQR], 57–70 years). A total of 211 patients (20.25%) experienced AF recurrence, with a median follow-up duration of 27.00 months (IQR, 17.00–42.00 months). The median CALLY index was 5.22 (IQR, 2.11–10.72). Compared to patients with a lower CALLY index, those with a higher CALLY index had lower CHA2DS2-VASc scores, smaller left atrial diameters, higher albumin levels, and lower SII levels. Baseline characteristics are summarized in Table 1.

**Table 1:** Characteristics of the subjects.

Variables		Cally I 0.86 (0.46,1.49) (n = 261)	Cally II 3.36 (2.75,4.25) (n = 260)	Cally III 7.35 (6.26,8.65) (n = 260)	Cally IV 15.76 (13.00,22.08)(n = 261)	Statistic	*P*
Age (years), M (Q_1_, Q_3_)	65.00 (57.00, 70.00)	68.00 (61.00,72.00)	64.00 (57.00,70.00)	62.00 (55.00,69.00)	64.00 (55.00,69.00)	χ^2^ = 36.67	**<.001**
Gender, n (%)						χ^2^ = 8.96	**.030**
Female	377 (36.18)	92 (35.25)	100 (38.46)	108 (41.54)	77 (29.50)		
Male	665 (63.82)	169 (64.75)	160 (61.54)	152 (58.46)	184 (70.50)		
BMI(kg/m^2^), M (Q_1_, Q_3_)	24.64 (22.66, 26.77)	24.50 (22.31,26.94)	24.80 (22.89,27.11)	24.92 (23.39,27.15)	24.51 (22.05,26.12)	χ^2^ = 10.36	**.016**
AF Duration(months), M (Q_1_, Q_3_)	12.00 (1.00, 36.00)	12.00 (1.00,36.00)	12.00 (1.00,36.00)	12.00 (1.38,36.00)	9.00 (1.00,36.00)	χ^2^ = 1.09	.781
Creatine(umol/L), Mean ± SD	77.64 ± 31.10	83.77 ± 53.81	74.74 ± 15.84	76.03 ± 17.97	76.01 ± 18.81	F = 4.66	**.003**
Platelet counts(10^9^/L), Mean ± SD	203.03 ± 58.69	202.94 ± 73.16	200.05 ± 54.58	203.81 ± 51.38	205.33 ± 53.16	F = 0.37	.774
TC(mmol/L), Mean ± SD	4.82 ± 6.52	6.49 ± 12.73	4.30 ± 1.38	4.34 ± 1.10	4.17 ± 1.12	F = 7.72	**<.001**
LDL-C(mmol/L), Mean ± SD	2.36 ± 1.96	2.58 ± 3.69	2.27 ± 0.72	2.38 ± 0.80	2.22 ± 0.74	F = 1.75	.155
Left Atrial Diameter(mm), M (Q_1_, Q_3_)	39.00 (34.00, 43.00)	41.00 (36.10,46.00)	39.00 (34.41,43.00)	38.00 (33.30,43.00)	37.00 (33.00,41.00)	χ^2^ = 48.83	**<.001**
SBP(mm Hg), M (Q_1_, Q_3_)	128.00 (115.00, 140.00)	126.00 (115.00,140.00)	128.00 (114.00,143.50)	128.00 (118.00,140.00)	127.00 (115.00,140.00)	χ^2^ = 2.23	0.526
DBP(mm Hg), M (Q_1_, Q_3_)	79.00 (71.00, 87.00)	80.00 (71.00,88.00)	79.00 (70.00,87.00)	78.00 (71.00,86.00)	79.00 (71.00,85.00)	χ^2^ = 2.64	.451
CHA2DS2 VASc, M (Q_1_, Q_3_)	2.00 (1.00, 3.00)	3.00 (2.00,4.00)	2.00 (1.00,3.00)	2.00 (1.00,3.00)	2.00 (1.00,3.00)	χ^2^ = 46.41	**<.001**
HAS-BLED, M (Q_1_, Q_3_)	1.00 (0.00, 1.00)	1.00 (0.00,1.00)	1.00 (0.00,1.00)	1.00 (0.00,1.00)	1.00 (0.00,1.00)	χ^2^ = 11.93	**0.008**
NTproBNP(pg/mL), M (Q_1_, Q_3_)	141.46 (61.00, 384.38)	250.00 (121.00,558.00)	141.46 (61.79,363.25)	120.50 (46.67,309.15)	119.00 (46.00,262.00)	χ^2^ = 50.43	**<.001**
eGFR(mL/min/1.73m2), M (Q_1_, Q_3_)	89.00 (77.00, 99.00)	86.00 (73.00,95.00)	89.50 (78.00,98.00)	89.00 (78.00,99.00)	90.00 (78.00,103.00)	χ^2^ = 17.49	**<.001**
Triglyceride(mmol/L), M (Q_1_, Q_3_)	1.20 (0.89, 1.64)	1.20 (0.87,1.63)	1.20 (0.96,1.71)	1.20 (0.87,1.65)	1.20 (0.86,1.58)	χ^2^ = 4.76	.190
LVEF(%), M (Q_1_, Q_3_)	62.00 (57.00, 66.00)	60.00 (53.00,65.00)	62.00 (56.00,66.23)	63.00 (58.00,66.00)	62.76 (58.00,67.00)	χ^2^ = 22.51	**<.001**
HDL(mmol/L), M (Q_1_, Q_3_)	1.16 (1.00, 1.35)	1.14 (0.96,1.32)	1.13 (1.01,1.33)	1.17 (0.99,1.37)	1.20 (1.04,1.37)	χ^2^ = 5.32	.150
Albumin(g/L), M (Q_1_, Q_3_)	40.20 (37.50, 43.50)	38.70 (36.10,41.70)	40.70 (37.88,43.95)	40.65 (38.08,43.62)	41.00 (37.80,44.30)	χ^2^ = 38.40	**<.001**
HbA1C, M (Q_1_, Q_3_)	6.00 (5.70, 6.35)	6.10 (5.81,6.50)	6.00 (5.76,6.30)	5.99 (5.70,6.30)	5.90 (5.70,6.20)	χ^2^ = 32.94#	**<.001**
SurvivalTime (months), M (Q_1_, Q_3_)	27.00 (17.00, 42.00)	22.00 (13.00,37.00)	27.00 (17.75,43.00)	29.00 (18.00,44.00)	29.00 (19.00,44.00)	χ^2^ = 20.81	**<.001**
Percentage of lymphocytes, M (Q_1_, Q_3_)	29.60 (23.90, 35.45)	24.40 (19.30,30.20)	28.45 (23.03,33.80)	31.60 (26.58,35.82)	33.20 (28.50,38.40)	χ^2^ = 138.71	**<.001**
WBC(*10^9^/L), M (Q_1_, Q_3_)	5.75 (4.90, 6.90)	6.20 (5.10,7.50)	5.70 (4.90,6.82)	5.60 (4.80,6.70)	5.70 (4.90,6.60)	χ^2^ = 14.79	**.002**
Neutrophil counts(*10^9^/L), M (Q_1_, Q_3_)	3.40 (2.70, 4.30)	4.00 (3.00,5.10)	3.40 (2.70,4.30)	3.30 (2.70,4.10)	3.20 (2.50,3.90)	χ^2^ = 50.51	**<.001**
Lymphocyte counts(*10^9^/L), M (Q_1_, Q_3_)	1.70 (1.30, 2.10)	1.50 (1.20,1.80)	1.60 (1.30,2.00)	1.70 (1.40,2.20)	1.80 (1.50,2.20)	χ^2^ = 70.46	**<.001**
MCV, M (Q_1_, Q_3_)	92.10 (89.50, 94.80)	92.10 (89.50,94.90)	92.00 (89.38,95.10)	91.90 (89.47,94.40)	92.30 (89.60,94.70)	χ^2^ = 0.68	.879
RDW, M (Q_1_, Q_3_)	12.70 (12.40, 13.20)	12.90 (12.50,13.50)	12.70 (12.40,13.20)	12.70 (12.30,13.12)	12.60 (12.20,13.10)	χ^2^ = 35.50	**<.001**
ALT(U/L), M (Q_1_, Q_3_)	21.00 (15.00, 30.00)	22.00 (14.00,34.00)	21.00 (16.00,30.25)	21.00 (14.00,30.25)	20.00 (15.00,28.00)	χ^2^ = 2.76	.430
AST(U/L), M (Q_1_, Q_3_)	24.00 (20.00, 29.00)	25.00 (20.00,32.00)	24.00 (20.00,29.00)	24.00 (20.00,28.00)	22.00 (20.00,28.00)	χ^2^ = 11.47	**.009**
ALP(U/L), M (Q_1_, Q_3_)	75.00 (62.00, 90.00)	81.00 (67.00,97.00)	78.00 (65.00,93.00)	72.50 (61.00,88.00)	69.00 (58.00,82.00)	χ^2^ = 43.36	**<.001**
CRP(mg/L), M (Q_1_, Q_3_)	1.30 (0.70, 3.00)	7.30 (3.90,12.00)	2.00 (1.40,2.50)	1.00 (0.74,1.30)	0.40 (0.30,0.60)	χ^2^ = 877.73	**<.001**
CALLY, M (Q_1_, Q_3_)	5.22 (2.11, 10.72)	0.86 (0.46,1.49)	3.36 (2.75,4.25)	7.35 (6.26,8.65)	15.76 (13.00,22.08)	χ^2^ = 975.94	**<.001**
SII, M (Q_1_, Q_3_)	392.61 (285.02, 567.80)	498.46 (351.33,771.47)	438.03 (303.25,591.72)	371.48 (285.68,479.95)	340.05 (239.08,456.92)	χ^2^ = 83.86	**<.001**
NHR, M (Q_1_, Q_3_)	3.02 (2.24, 4.00)	3.47 (2.70,4.87)	2.99 (2.26,4.32)	2.98 (2.14,3.76)	2.69 (2.00,3.47)	χ^2^ = 52.41	**<.001**
PHR, M (Q_1_, Q_3_)	168.73 (133.33, 210.73)	171.56 (128.80,222.15)	167.85 (136.91,210.72)	165.35 (133.96,204.81)	170.45 (135.16,204.83)	χ^2^ = 0.13	.988
LHR, M (Q_1_, Q_3_)	1.43 (1.10, 1.91)	1.30 (0.97,1.69)	1.38 (1.05,1.92)	1.47 (1.16,1.95)	1.58 (1.21,1.94)	χ^2^ = 30.92	**<.001**
TyG, M (Q_1_, Q_3_)	8.55 (8.18, 8.90)	8.60 (8.17,9.01)	8.61 (8.26,8.92)	8.52 (8.18,8.92)	8.45 (8.14,8.75)	χ^2^ = 10.06	**.018**
Glucose(mmol/L), M (Q_1_, Q_3_)	5.19 (4.68, 5.82)	5.45 (4.83,6.12)	5.19 (4.71,5.81)	5.22 (4.71,5.75)	4.99 (4.50,5.46)	χ^2^ = 30.85	**<.001**
Status, n(%)						χ^2^ = 55.23	**<.001**
None	831 (79.75)	180 (68.97)	189 (72.69)	223 (85.77)	239 (91.57)		
Recurrence	211 (20.25)	81 (31.03)	71 (27.31)	37 (14.23)	22 (8.43)		
Type of AF, n(%)						χ^2^ = 24.31	**<.001**
Paroxysmal	594 (57.01)	122 (46.74)	143 (55.00)	152 (58.46)	177 (67.82)		
Persistent	448 (42.99)	139 (53.26)	117 (45.00)	108 (41.54)	84 (32.18)		
Combined with other arrythmias, n (%)						χ^2^ = 0.56	.906
None	871 (83.59)	216 (82.76)	221 (85.00)	216 (83.08)	218 (83.52)		
Yes	171 (16.41)	45 (17.24)	39 (15.00)	44 (16.92)	43 (16.48)		
Hypertension, n (%)						χ^2^ = 6.53	.089
None	508 (48.75)	116 (44.44)	120 (46.15)	129 (49.62)	143 (54.79)		
Yes	534 (51.25)	145 (55.56)	140 (53.85)	131 (50.38)	118 (45.21)		
Coronary artery disease, n (%)						χ^2^ = 7.26	.064
None	905 (86.85)	218 (83.52)	220 (84.62)	234 (90.00)	233 (89.27)		
Yes	137 (13.15)	43 (16.48)	40 (15.38)	26 (10.00)	28 (10.73)		
Diabetes, n (%)						χ^2^ = 12.19	**.007**
None	900 (86.37)	209 (80.08)	227 (87.31)	232 (89.23)	232 (88.89)		
Yes	142 (13.63)	52 (19.92)	33 (12.69)	28 (10.77)	29 (11.11)		
the use of class I antiarrhythmic drugs, n (%)						χ^2^ = 0.50	.919
None	906 (86.95)	230 (88.12)	224 (86.15)	225 (86.54)	227 (86.97)		
Yes	136 (13.05)	31 (11.88)	36 (13.85)	35 (13.46)	34 (13.03)		
the use of class II antiarrhythmic drugs, n (%), n (%)						χ^2^ = 1.78	.620
None	729 (69.96)	175 (67.05)	185 (71.15)	181 (69.62)	188 (72.03)		
Yes	313 (30.04)	86 (32.95)	75 (28.85)	79 (30.38)	73 (27.97)		
the use of class III antiarrhythmic drugs, n (%)						χ^2^ = 8.67	**.034**
None	303 (29.08)	74 (28.35)	75 (28.85)	92 (35.38)	62 (23.75)		
Yes	739 (70.92)	187 (71.65)	185 (71.15)	168 (64.62)	199 (76.25)		

F: ANOVA, #: Kruskal–waills test, χ^2^: Chi-square test.

SD = standard deviation, M = Median, Q_1_ = 1st Quartile, Q_3_ = 3st Quartile.

### 3.2. Heatmap of correlations among various inflammatory markers

**Figure 2.**
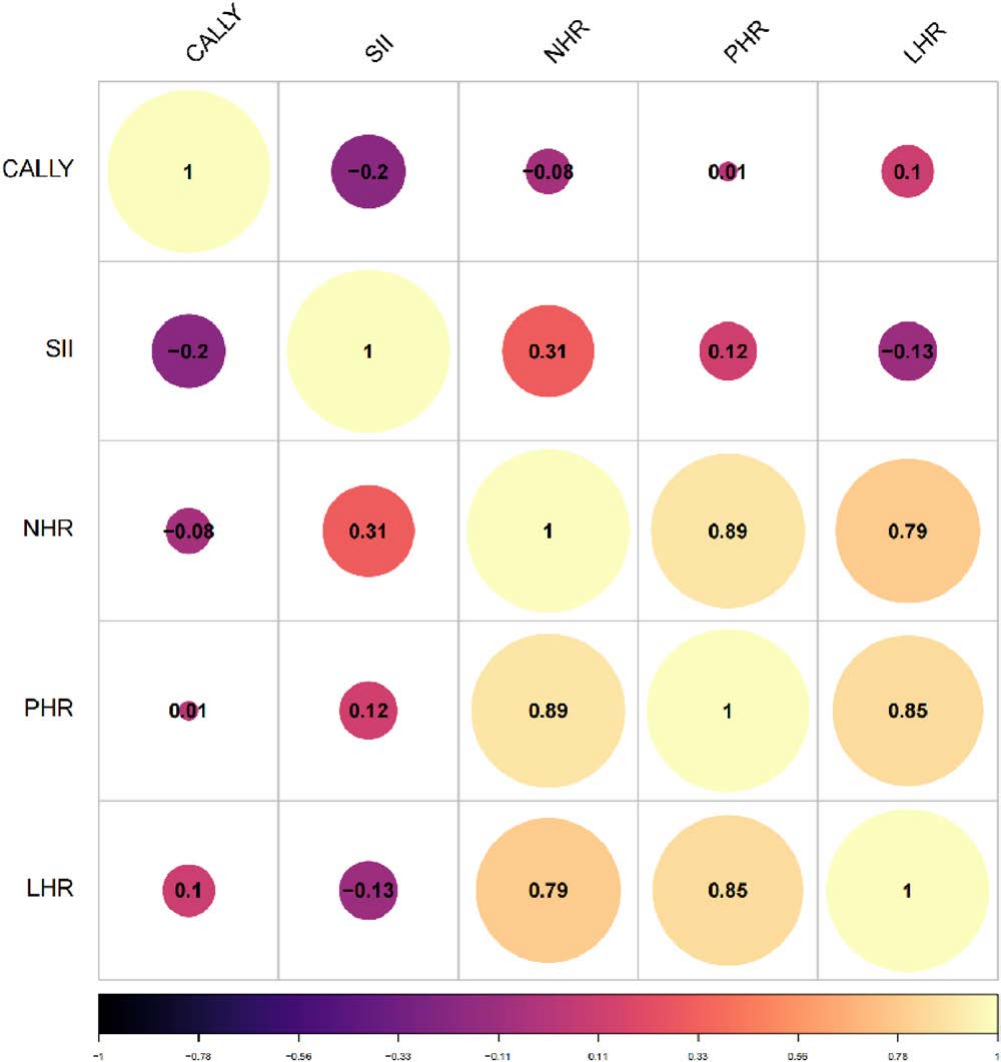
Correlation heatmap among inflammatory markers.

The heatmap (Fig. 2) reveals a negative correlation between the CALLY index and traditional systemic immune-inflammatory markers, indicating that a higher CALLY index is associated with a lower degree of systemic immune inflammation.

### 3.3. Univariate Cox regression to identify risk factors associated with atrial fibrillation recurrence

Table 2 identifies type of AF, AF duration, NT-proBNP, triglycerides, left atrial diameter, LVEF, HbA1c, TyG index, glucose, and the use of class II antiarrhythmic drugs as risk factors for AF recurrence. Compared to patients with paroxysmal AF, those with persistent AF have a 1.07-fold increased hazard of AF recurrence (hazard ratio [HR]: 2.07, 95% confidence interval [CI]: 1.57–2.72, *P* < .001). In patients using class II antiarrhythmic drugs, the hazard of AF recurrence is 0.55 times higher compared to those not using these medications (HR = 1.55, 95% CI: 1.17–2.04, *P* = .002). For each unit increase in AF duration, the hazard of recurrence increases by 1% (HR = 1.01, 95% CI: 1.01–1.01, *P* = .003). For each unit increase in NT-proBNP, the hazard of recurrence also increases by 1% (HR = 1.01, 95% CI: 1.01–1.01, *P* < .001). Each unit increase in triglycerides results in a 16% higher hazard of recurrence (HR = 1.16, 95% CI: 1.01–1.34, *P* = .033). For each unit increase in left atrial diameter, the hazard of recurrence increases by 7% (HR = 1.07, 95% CI: 1.05–1.09, *P* < .001). Each unit increase in LVEF reduces the hazard of recurrence by 2% (HR = 0.98, 95% CI: 0.97–0.99, *P* = .004). Each unit increase in HbA1c raises the hazard of recurrence by 14% (HR = 1.14, 95% CI: 1.01–1.30, *P* = .036). Each unit increase in TyG index increases the hazard of recurrence by 27% (HR = 1.27, 95% CI: 1.01–1.61, *P* = .048), and each unit increase in glucose increases the hazard of recurrence by 8% (HR = 1.08, 95% CI: 1.01–1.16, *P* = .047).

**Table 2:** Univariate Cox regression.

Variables	β	S.E	Z	*P*	HR (95%CI)
Gender					
Female					1.00 (Reference)
Male	0.16	0.15	1.05	.292	1.17 (0.87–1.56)
Type of AF					
Paroxysmal					1.00 (Reference)
Persistent	0.73	0.14	5.18	**<.001**	2.07 (1.57–2.72)
Combined with other arrythmias					
None					1.00 (Reference)
Yes	0.08	0.18	0.44	.664	1.08 (0.76–1.54)
Hypertension					
None					1.00 (Reference)
Yes	0.15	0.14	1.06	.288	1.16 (0.88–1.52)
Coronary artery disease					
None					1.00 (Reference)
Yes	0.12	0.20	0.58	.559	1.12 (0.76–1.65)
Diabetes					
None					1.00 (Reference)
Yes	0.14	0.19	0.71	.478	1.15 (0.78–1.68)
The use of class I antiarrhythmic drugs					
None					1.00 (Reference)
Yes	0.12	0.19	0.62	.537	1.12 (0.77–1.63)
The use of class II antiarrhythmic drugs					
None					1.00 (Reference)
Yes	0.44	0.14	3.07	**.002**	1.55 (1.17–2.04)
The use of class III antiarrhythmic drugs					
None					1.00 (Reference)
Yes	0.01	0.15	0.06	.956	1.01 (0.75–1.36)
Age	0.01	0.01	1.49	.136	1.01 (1.00–1.03)
CHA2DS2-VASc	0.06	0.05	1.33	.182	1.06 (0.97–1.16)
HAS-BLED	0.13	0.08	1.72	.086	1.14 (0.98–1.32)
AF duration	0.01	0.00	2.96	**.003**	1.01 (1.01–1.01)
NTproBNP	0.01	0.00	3.47	**<.001**	1.01 (1.01–1.01)
eGFR	-0.00	0.00	−0.46	.643	1.00 (0.99–1.01)
Triglyceride	0.15	0.07	2.13	**.033**	1.16 (1.01–1.34)
BMI	0.01	0.02	0.48	.631	1.01 (0.97–1.06)
Left Atrial Diameter	0.07	0.01	6.86	**<.001**	1.07 (1.05–1.09)
LVEF	−0.02	0.01	−2.89	**.004**	0.98 (0.97–0.99)
HDL	−0.47	0.24	−1.94	.053	0.62 (0.39–1.01)
Albumin	−0.02	0.02	−1.13	.259	0.98 (0.95–1.01)
Creatine	0.00	0.00	0.97	.332	1.00 (1.00–1.00)
HbA1C	0.14	0.06	2.10	**.036**	1.14 (1.01–1.30)
TyG	0.24	0.12	1.97	**.048**	1.27 (1.01–1.61)
Glucose	0.07	0.04	1.99	**.047**	1.08 (1.01–1.16)
TC	−0.01	0.01	−0.43	.664	0.99 (0.97–1.02)
LDL C	0.02	0.04	0.46	.647	1.02 (0.94–1.10)
ALT	−0.00	0.00	−0.82	.414	1.00 (0.99–1.00)
AST	0.00	0.00	0.74	.457	1.00 (0.99–1.01)
ALP	0.00	0.00	1.16	.247	1.00 (1.00–1.01)

CI = confidence interval, HR = hazard ratio.

### 3.4. Multivariate Cox regression with multiple models to compare the associations of different inflammatory indices with atrial fibrillation recurrence

In the multivariate Cox regression analysis (Table 3), after adjusting for confounding variables (including AF type, AF duration, NT-proBNP, triglycerides, left atrial diameter, LVEF, HbA1c, TyG index, glucose, and the use of class II antiarrhythmic drugs), the CALLY index was identified as a protective factor against AF recurrence. For each 1-unit increase in the CALLY index, the hazard of AF recurrence decreased by 10% (HR = 0.8974, 95% CI: 0.8674–0.9284, *P* < .001).

**Table 3:** Multiivariate Cox regression.

Variables	Model 1		Model 3			
	HR (95% CI)	*P*	HR (95% CI)	*P*	HR (95% CI)	*P*
CALLY	0.9035 (0.8760–0.9319)	**<.0001**	0.8924 (0.8626–0.9232)	**<.0001**	0.8974 (0.8674–0.9284)	**<.0001**
SII	0.9998 (0.9995–1.0002)	.3691	0.9998 (0.9991–1.0005)	.5491	0.9998 (0.9992–1.0005)	.6007
NHR	0.9870 (0.9311–1.0462)	.6596	0.9087 (0.7868–1.0495)	.1925	0.9031 (0.7827–1.0421)	.1629
PHR	0.9986 (0.9966–1.0007)	.1881	0.9982 (0.9953–1.0011)	.2327	0.9981 (0.9950–1.0011)	.2152
LHR	0.9592 (0.8169–1.1264)	.6113	1.2877 (0.9128–1.8166)	.1499	1.2215 (0.8559–1.7432)	.2702

CI = confidence interval, HR = hazard ratio.

Model1: Crude.

Model2: Adjust: Age, Sex, BMI.

Model3: Adjust: Age, Sex, BMI,Type of AF, II, AF Duration, Left atrial diameter, NTproBNP, Triglyceride, LVEF, HbA1C, TyG, Glucose.

### 3.5. Restricted cubic spline analysis of the CALLY index and atrial fibrillation recurrence

**Figure 3.**
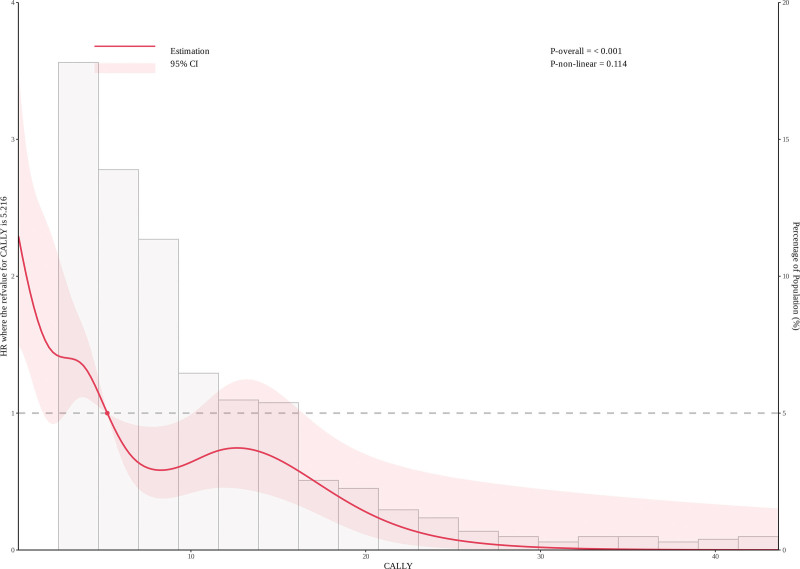
The restrictive cubic spline plot of the CALLY Index.

To further clarify the relationship between the CALLY index and AF recurrence, we constructed the RCS curve shown in the Figure 3. The analysis reveals a nonlinear association between the CALLY index and AF recurrence (*P* overall < .001).

### 3.6. Subgroup analysis

**Figure 4.**
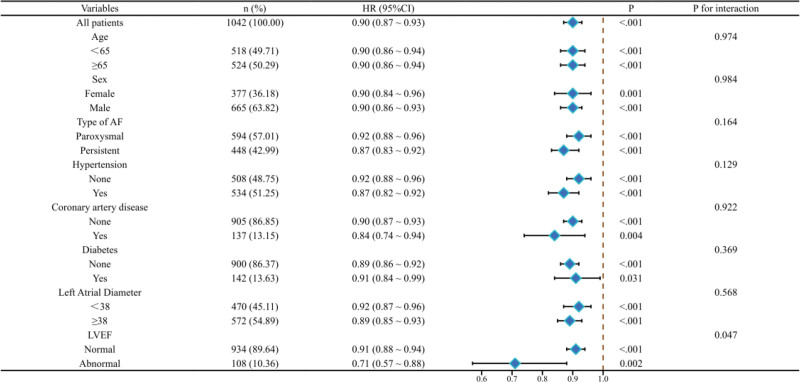
Subgroup forest plot.

In the subgroup analysis (Fig. 4), we observed that the effect of the CALLY index on AF recurrence was consistent across all subgroups, mirroring the overall trend: a higher CALLY index was associated with a lower hazard of AF recurrence. Notably, in the LVEF abnormal subgroup, there was an interaction between the CALLY index and AF recurrence (HR = 0.71, 95% CI: 0.57–0.88, *P* = .002, *P* for interaction = .047).

### 3.7. Predictive value of various inflammatory markers

**Figure 5.**
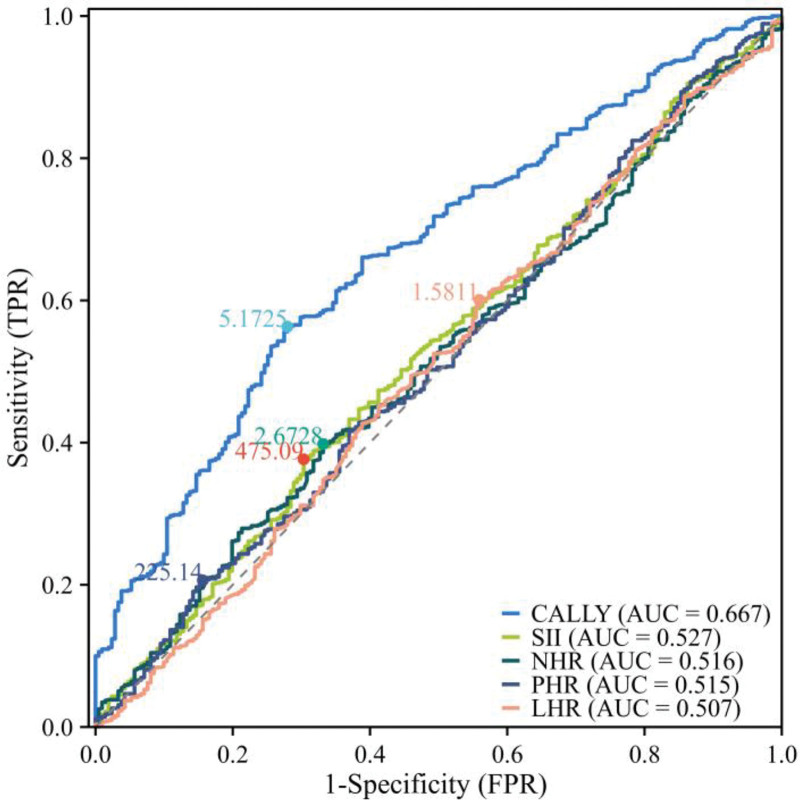
Predictive value of inflammatory indices for atrial fibrillation recurrence.

To evaluate the predictive value of different immune-inflammatory markers for AF recurrence, we constructed ROC curves (Fig. 5) and found that the CALLY index demonstrated superior predictive value compared to traditional immune-inflammatory markers (AUC = 0.667, 95% CI: 0.628–0.706).

## 4. Discussion

Our findings suggest that the CALLY index is specifically associated with the prognosis of AF RFCA. An increase in the CALLY index is linked to a reduced hazard of AF recurrence, and the relationship between the 2 is overall nonlinear. Furthermore, the CALLY index demonstrates superior predictive value for AF recurrence compared to traditional immune-inflammatory markers such as SII, NHR, PHR, and LHR.

The CALLY index integrates CRP, serum albumin, and lymphocyte count, markers of inflammation, nutritional status, and immune function, respectively. While conventional inflammatory markers such as SII, NHR, and PHR are readily available in clinical practice and offer valuable predictive information for cardiovascular events, they did not demonstrate prognostic utility for AF ablation outcomes in our cohort study.^[12,13]^ This suggests that AF recurrence is a multifactorial process requiring a more comprehensive and multidimensional assessment. Given the complex interplay among inflammation, nutrition, and immunity in influencing AF ablation prognosis, the CALLY index, by synthesizing these 3 key dimensions, provides a more holistic evaluation. Therefore, we will examine the impact of the CALLY index on AF ablation outcomes through the lenses of inflammation, nutritional status, and immune function.

CRP is an affordable and widely accessible clinical marker that has been consistently validated in large-scale studies as a practical and reproducible predictor of AF onset.^[14,15]^ CRP levels are typically higher in AF patients than in those maintaining sinus rhythm, and among AF patients, those with persistent AF exhibit higher CRP levels than those with paroxysmal AF. Elevated CRP has also been identified as a predictor of new-onset AF.^[14–16]^ The prognostic relevance of CRP in AF ablation can be attributed to several mechanisms:

CRP serves as a systemic inflammatory marker, with elevated levels indicating an enhanced inflammatory state that increases susceptibility to AF. Inflammation promotes atrial remodeling and can trigger atrial arrhythmias, perpetuating a cycle of “AF begetting AF.”^[4,17]^ Kallergis et al observed that CRP levels in patients with AF recurrence remained similar to pre-ablation levels.^[18]^ Additionally, electrical and fibrotic remodeling in AF can activate epigenetic regulation; as noted by Sardu C et al., miRNA activation and expression patterns are associated with these adaptive processes.^[19]^

Elevated serum CRP can shorten the atrial effective refractory period and induce calcium overload in atrial myocytes, potentially leading to cardiomyocyte death.^[20]^ This process may trigger the release of damage-associated molecular patterns, which are recognized by Toll-like receptors. Subsequent activation of NF-κB and AP-1 transcription factors upregulates pro-inflammatory genes, including cytokines and chemokines, initiating a low-grade inflammatory response aimed at tissue repair.^[4]^ While such inflammation is initially protective, chronic activation results in reactive oxygen species and cytokine production, promoting atrial fibrosis, hypertrophy, and apoptosis. These changes worsen the atrial substrate and disrupt electrical conduction, thereby sustaining AF.

CRP levels correlate with oxidative stress, which plays a key role in atrial remodeling pathophysiology.^[21–23]^

In our study, lower CALLY index values corresponded to higher CRP levels, indicating that patients with AF recurrence likely presented with a heightened inflammatory state prior to ablation.Albumin reflects the nutritional status of the body, and our previous research has demonstrated a relationship between albumin levels and AF recurrence, a finding also confirmed by Schamroth et al.^[24,25]^ As the most abundant protein in human serum, albumin plays several vital roles, including anti-inflammatory, antioxidant, anticoagulant, antiplatelet aggregation, and maintaining colloid osmotic pressure, as well as transporting various endogenous and exogenous substances.^[26,27]^ Studies have suggested that albumin contains a high concentration of mercaptan groups, which make up about 80% of plasma’s total mercaptan content, playing a key role in scavenging reactive oxygen species and nitrogen, as well as carrying nitric oxide (NO), thereby exerting potent antioxidant activity.^[28]^ This antioxidant and anti-inflammatory effect may contribute to protecting atrial myocytes against fibrosis in patients with AF.^[29,30]^ Furthermore, hypoalbuminemia serves as a marker of an exacerbated inflammatory state. In clinical settings, heart failure patients often present with hypoalbuminemia. Our study found that the CALLY index showed an interaction in predicting AF recurrence in heart failure patients. This is likely due to the reduced cardiac output in heart failure, which leads to tissue hypoxia. Additionally, hepatic congestion further impairs albumin synthesis, resulting in a poorer prognosis for AF ablation patients in this population.

Lymphocytes have been shown to be associated with AF, with lymphocyte count reflecting the level of systemic inflammatory activity.^[4,7]^ The interplay of different lymphocyte subtypes plays a protective role in AF, as “immune remodeling” is involved throughout the development and maintenance of the arrhythmia. Various lymphocyte subsets, including CD4 + (Th1 or Th2 cells), CD8 + T cells, natural killer cells, regulatory T cells, and B lymphocytes, contribute differently to chronic inflammation.^[31,32]^ Studies have shown that in patients with atrial myopathy, lymphomononuclear cells infiltrate the atrial tissue, where they secrete high levels of TNF, TGF-β1, and IL-6.^[33]^ These cytokines subsequently promote atrial fibrosis and electrical remodeling. Cellular infiltration plays a functional role in cardiac fibrosis, a process highly dependent on the cardiac environment and T-cell subtypes. B lymphocytes, primarily through antibody secretion, can become pathologically activated and produce autoantibodies. Both B and T cells are involved in the atrial remodeling process. Ultimately, inappropriate immune-inflammatory damage leads to the deterioration of the atrial substrate, contributing to the high recurrence rate of AF.

In this study, we propose that AF recurrence is closely related to the interplay between pre-ablation inflammation, nutritional status, and immune function. Elevated CRP levels activate inflammatory cytokines such as IL-1, IL-6, and TNF-α, which accelerate metabolic processes and nutrient consumption in atrial myocytes, leading to nutritional imbalance at the cellular level. Inflammation also triggers an immune response within atrial myocytes, activating protective mechanisms that promote atrial fibrosis. This not only contributes to atrial structural remodeling but also disrupts electrophysiological conduction, thereby worsening the atrial substrate and ultimately reducing ablation efficacy. Additionally, these processes can impair calcium channel function, leading to autonomic dysregulation in AF patients and further destabilizing atrial electrical activity.^[34]^ In summary, the interaction among inflammation, nutrition, and immunity forms a complex vicious cycle that accelerates the progression of AF. While ablation effectively addresses electrical remodeling, it does not reverse the underlying atrial substrate. This highlights the importance of implementing clinical interventions to reduce inflammation levels in AF patients before ablation.

Factors such as AF type, AF duration, NT-proBNP, triglycerides, left atrial diameter, LVEF, HbA1c, TyG, and glucose have been well-established as risk factors for AF recurrence.^[1,3,17,35]^ Our findings further support that the CALLY index provides a reliable reflection of the immune-inflammatory state in AF ablation patients, and it holds predictive value for AF ablation outcomes.

### 4.1. Limitations

This study inevitably has several limitations. First, although it is based on data from 2 centers, the sample size remains relatively small, and the findings may need to be validated in larger cohorts to improve generalizability. Second, as a retrospective cohort study, the causal relationship between the CALLY index and AF recurrence remains uncertain. Moreover, we only considered the preoperative Cally levels to guide reasonable clinical interventions for patients scheduled for ablation based on their preprocedural immune, inflammatory, and nutritional status. We did not account for the dynamic changes in Cally or address postoperative variations in inflammatory levels in atrial fibrillation patients. It remains unclear how the immune, inflammatory, and nutritional status following AF ablation may influence atrial substrate remodeling. Third, our analysis focuses on the macroscopic clinical aspects of inflammation, nutrition, and immune function in relation to AF ablation outcomes, without delving into the molecular or cellular mechanisms underlying these complex relationships. Fourth, while anti-inflammatory interventions have been shown to improve AF ablation outcomes, our findings are inconsistent with results from randomized controlled trials, likely due to the limitations inherent in small sample, retrospective cohort studies. Finally, post-ablation management of AF is multifaceted, and evaluating the prognosis of AF patients solely based on the CALLY index is overly simplistic. A comprehensive approach that incorporates AF type, left atrial diameter, and other risk factors is needed for more accurate prognostic assessment.

## 5. Conclusion

Our study demonstrates that the CALLY index is associated with AF recurrence and highlights the complex interactions between inflammation, immune response, and nutritional status in its pathogenesis. Moreover, we found that higher pre-ablation CALLY index values are associated with better ablation outcomes, offering valuable clinical guidance for the management of AF RFCA.

## Acknowledgments

We sincerely thank all team members for their dedicated efforts and acknowledge the statistical analysis support provided by Zstats 1.0 (www.zstats.net).

## Author contributions

**Conceptualization:** Xiaojian Zhang.

**Data curation:** Qingru Zhu.

**Formal analysis:** Xiaojian Zhang, Yanping Yin, Sixiang Jia.

**Funding acquisition:** Yanping Yin, Sixiang Jia, Shudong Xia.

**Investigation:** Qingru Zhu.

**Methodology:** Xiaojian Zhang.

**Project administration:** Weili Ge, Shudong Xia.

**Resources:** Xiaojian Zhang, Yanping Yin, Shudong Xia.

**Software:** Sixiang Jia.

**Supervision:** Sixiang Jia, Weili Ge.

**Validation:** Qingru Zhu.

**Writing – original draft:** Xiaojian Zhang.

**Writing – review & editing:** Weili Ge, Shudong Xia.






