# Supplementary material for: Association between C-reactive protein-albumin-lymphocyte (CALLY) index and atrial fibrillation recurrence: A retrospective cohort study
Source: Medicine (Baltimore). 2026 May 22;105(21):e49012. doi: 10.1097/MD.0000000000049012 (PMC13200956; doi:10.1097/MD.0000000000049012)
Supplement: Supplementary file 1 [file medi-105-e49012-s001.docx]

**Supplementary A1. Radiofrequency Catheter Ablation Procedure for Atrial Fibrillation**

The Radiofrequecy Catheter Ablation (RFCA) procedure was conducted under sedation, with continuous monitoring of ECG and oxygen saturation throughout the ablation process. Transesophageal cardiac ultrasound was carried out 24 h before RFCA to rule out the presence of left atrial thrombus. Standardized circumflex pulmonary vein isolation was performed on all patients. Intraoperatively, a sheath was inserted through the femoral vein, and after accessing the left atrium via septal puncture, the Carto 3 three-dimensional anatomical mapping system was utilized for precise localization during circumflex pulmonary vein isolation. Ablation power ranged from 35-45 W for the anterior wall and 30-40 W for the posterior wall. Additional ablation pathways, such as superior vena cava isolation, roof line or mitral isthmus line ablation of the left atrium, and tricuspid isthmus ablation, were determined by the operator based on individual requirements. Pulmonary venous potentials were recorded using a LASSO® NAV catheter before, during, and after the ablation procedure to assess its effectiveness. The ablation endpoint was achieved when the bi-directional transmission (afferent and efferent) of pulmonary venous potentials ceased completely for at least 30 min. In cases of persistent atrial fibrillation or other atrial tachycardia post-ablation, direct current cardioversion could be considered as an option to restore sinus rhythm.
