# Supplementary material for: Association between C-reactive protein-albumin-lymphocyte (CALLY) index and atrial fibrillation recurrence: A retrospective cohort study
Source: Medicine (Baltimore). 2026 May 22;105(21):e49012. doi: 10.1097/MD.0000000000049012 (PMC13200956; doi:10.1097/MD.0000000000049012)
Supplement: Supplementary file 2 [file medi-105-e49012-s002.docx]

**Supplementary A2. Postablation follow-up of atrial fibrillation**

Following ablation, all patients undergo outpatient follow-up and 24-hour ambulatory electrocardiogram monitoring at 1, 3, and 6 months post-procedure. At 12 months post-procedure, outpatient follow-up and 7-day long-term ambulatory electrocardiogram monitoring are conducted. Subsequently, outpatient follow-up and 24-hour ambulatory electrocardiogram monitoring are performed every 6 months.

Sequential oral anticoagulant therapy should be initiated the day after RFCA to minimize the interruption of anticoagulation treatment. According to individual CHA2DS2-VASc scores, AF patients are advised to continue oral anticoagulants for a minimum of two months. Persistent AF patients are prescribed amiodarone for three months post-RFCA to uphold sinus rhythm and undergo regular monitoring for medication side effects. If there is no recurrence during the three-month blanking period, patients with paroxysmal AF should refrain from using class I or III antiarrhythmic drugs. In the event of AF recurrence, β-receptor blockers are prioritized as the first-line rate control treatment, with propafenone being considered if symptoms persist despite β-receptor blocker usage. All class I or III antiarrhythmic drugs are ceased at the conclusion of the blanking period.
