# Supplementary material for: Association between C-reactive protein-albumin-lymphocyte (CALLY) index and atrial fibrillation recurrence: A retrospective cohort study
Source: Medicine (Baltimore). 2026 May 22;105(21):e49012. doi: 10.1097/MD.0000000000049012 (PMC13200956; doi:10.1097/MD.0000000000049012)

**Supplementary A3. Calculation of CHA2DS2-VASc, HAS-BLED Scores and TyG**

The CHA2DS2-VASc score is utilized to estimate the risk of stroke in patients with atrial fibrillation (AF). The score is calculated by assigning points for the presence of specific risk factors: Congestive Heart Failure/Left Ventricular Dysfunction: 1 point, Hypertension: 1 point, Age 75 years or older: 2 points, Diabetes Mellitus: 1 point, Stroke/Transient Ischemic Attack (TIA)/Thromboembolism: 2 points, Vascular Disease (e.g., prior myocardial infarction, peripheral artery disease, aortic plaque): 1 point,Age 65-74 years: 1 point.

The HAS-BLED score is used to assess bleeding risk in patients with atrial fibrillation on anticoagulation therapy. It is calculated by adding points for the following factors:

Hypertension (H): Systolic BP >160 mmHg (1 point)

Abnormal renal function (A):

Serum creatinine >200 µmol/L or dialysis (2 points)

Mild renal impairment (1 point)

Abnormal liver function (B):

Cirrhosis or liver failure (3 points)

Mild liver disease (1 point)

Stroke (S): History of stroke or TIA (1 point)

Bleeding history (B): Previous major bleeding (1 point)

Labile INRs (L): Unstable or poorly controlled INR (1 point)

Elderly (E): Age >65 years (1 point)

Drugs or alcohol (D): Antiplatelets, NSAIDs, or alcohol abuse (1 point)

The total score ranges from 0 to 9. A higher score indicates a higher bleeding risk, with a score of 3 or more suggesting a need for closer monitoring of anticoagulation therapy.


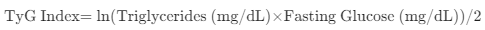

Supplement: Supplementary file 3 [file medi-105-e49012-s003.docx]
